# Chiral Liquid Crystal Lenses Confined in Microchannels

**DOI:** 10.3390/ma13173761

**Published:** 2020-08-26

**Authors:** Sean M. Hare, Beatrice Lunsford-Poe, MinSu Kim, Francesca Serra

**Affiliations:** Department of Physics and Astronomy, Johns Hopkins University, Baltimore, MD 21218, USA; share2@jhu.edu (S.M.H.); blunsfordpoe@umass.edu (B.L.-P.); mkim182@jhu.edu (M.K.)

**Keywords:** liquid crystals, micro-lenses, phase transition

## Abstract

It is known that the liquid crystalline smectic-A phase has geometric defects, called focal conic domains, which can be used as gradient-index microlenses. Cholesteric (chiral nematic) phases also have topological defects with a central symmetry and a singularity at their center. We explore a weakly chiral system in which both types of defects can be present in the same material at different temperatures, and with this strategy we create lenses whose focal length is tunable with temperature. We measure the focal length of the tunable lenses, and we investigate the behavior of the defects near the phase transition. We identify the experimental conditions that make the simultaneous presence of the smectic focal conic domains and the circular cholesteric domains possible, such as the concentration of chiral dopant and the rate of heating and cooling. The transformation of focal conic domains into circular cholesteric domains is a new example of memory at the phase transition between smectic-A and nematic liquid crystals.

## 1. Introduction

While liquid crystal (LC) technology is widely associated with displays and spatial light modulators [1,2], the LC potential for other optical elements has been only partly explored. In particular, LC-based lenses have shown increasing promise in the past years due to their potential for external actuation, low energy switching, polarization sensitivity, and tunability [3,4,5]. Moreover, LCs offer the possibility to create lenses in the micro-scale, which would otherwise require complex manufacturing.

LCs are fluids with orientational order, which can be changed and manipulated with external fields [6,7]. The simplest type of LC phases is the nematic phase. Here the rod-like molecules that compose the fluid have long-range orientational order, and their average orientation is defined by a headless vector called the nematic director. Most LC-based lenses are realized with nematic LC, and work because of the modulation of the nematic director orientation, which translates into a modulation of the refractive index [4,5]. Such modulation is induced by a combination of external fields and elastic energy associated to the molecular anchoring at the boundaries. These lenses rely on gentle modulations of the director; however, it is possible to create lenses utilizing discontinuities in the director field called topological defects. These defects are in fact characterized by a large change of the refractive index in their proximity [8]. Defects occur spontaneously in LCs, but they can also be created and manipulated through a combination of topological and topographical cues, surface anchoring and external fields [9,10]. For example, the so-called umbilic defects in nematic LCs can manipulate and focus light [11] or generate arrays for diffraction gratings [12].

Other LC phases have been explored for the construction of micro-lenses. In particular, defects of the smectic phase called focal conic domains (FCD) have shown interesting self-assembly and lensing properties [13,14]. Smectic phases are characterized by an arrangement of LC molecules in parallel molecular layers. Cholesteric, or chiral nematic, phases have also been used for tunable lenses [15]. In this phase the nematic director twists along one direction and creates a helical periodicity [6,16]. There are analogies between the smectic and the cholesteric phase, as both show a 1D periodic modulation. These analogies and differences are explored in [17]. Here, we are interested in topological defects of these two phases that can act as lenses.

**Figure 1:**
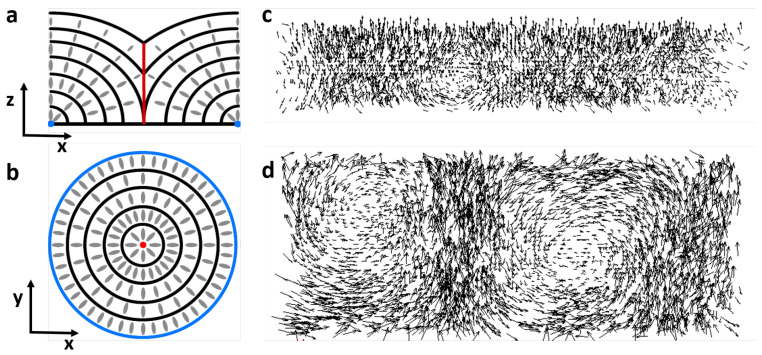
Circular domains in smectic and cholesteric liquid crystals (LCs). (**a**,**b**) Schematic of toric focal conic domains (FCDs), side view (**a**) and top view (**b**). The schematic highlights the smectic layers and the LC molecular arrangement. The central defect line is shown in red and the singular points at the edge of the defect are shown in blue. (**c**,**d**) Simulations of circular cholesteric domains, side view (**c**) and top view (**d**). The glyphs indicate the molecular arrangement. Computer simulations were performed minimizing the Landau–deGennes free energy [24] of chiral LCs confined in a channel with one homeotropic wall and three planar walls.

When confined in a thin film with antagonistic boundary conditions—planar on one side and perpendicular (homeotropic) on the other—smectic LCs form focal conic domains to minimize the total elastic energy while obeying the boundary conditions [18]. In FCDs, the singular points become arranged along an ellipse laying on the planar surface and a branch of hyperbola passing through the ellipse focus. When the elliptical defect has small eccentricity and the branch of hyperbola has very low curvature this structure approaches a toric FCD, such as the one schematized in Figure 1a,b. As the LC director bends towards the central defect line, it generates a gradient of refractive index from the edges of the defect to the center and focuses light at a distance that depends on the diameter of the FCD, the anchoring strength and the LC birefringence. FCDs can be arranged in linear arrays with appropriate boundary conditions [19].

Cholesteric LCs, on the other hand, can form circular domains with toroidal structures extensively studied in [20]. Figure 1c,d shows cross sections of the director field of these complex defect structures. Confinement favors the formation of such domains [21,22,23]. While the smectic focal conic domains are relatively temperature-independent, the cholesteric circular domains are more sensitive to temperature and to the pitch of the cholesteric helix. It is therefore tempting to think of a system in which two types of micro-lenses (temperature-sensitive and temperature-insensitive) can be created by self-assembly. However, the smectic phase and focal conic domains are incompatible with molecular twist, and high chirality suppresses the FCD formation. Therefore, we explore a system of smectic LCs to which a very small amount of chiral dopant is added. In this way, the smectic phase is preserved and relatively undisturbed, while the director field can still twist above the smectic-nematic phase transition.

Phase transitions are typically seen as processes that retain no previous information about the system. However, experiments with thin layers of LC at air-water interfaces have shown that the configuration of defects can be “remembered” going from the nematic to the smectic phase [25,26]. This phenomenon of geometric memory across phase transitions has been very little explored until recently. The focus of this research is to study tunable micro-lenses across the smectic and the cholesteric phase. We discuss which combinations of controlled temperature changes, chiral dopant concentrations, and confinements create lenses, whose locations are either correlated or completely unchanged across the phase transition. Geometric memory is not only a remarkable property but appears to be a driving force in the stability, tunability, and predictability of microlenses formation, all of which only become prominent as the chirality of the system is increased.

## 2. Results

Hybrid boundary conditions are created by using an air interface above (perpendicular anchoring) and SU-8 microchannel surfaces at the bottom (random planar alignment). Above 31 °C, the LC 8CB becomes nematic and the addition of chiral dopant creates a chiral nematic phase. CB15 concentrations are chosen such that the pitch (around 10 μm) is on the length scale of the width of the channels (40 μm and 50 μm). Further details are provided in the Materials and Methods section, including a schematic of the optical setup.

### 2.1. Lenses

**Figure 2:**
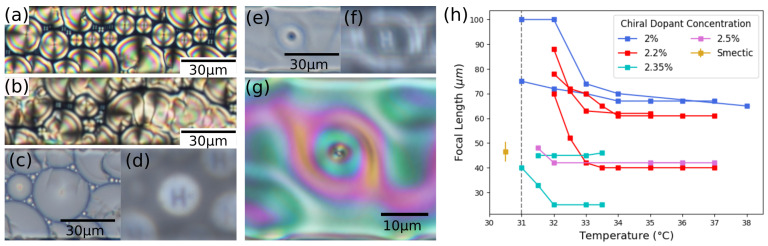
Lenses in smectic and cholesteric LC defects. (**a**,**b**) Smectic FCDs viewed between crossed polarizers. (**b**) Disrupted focal conic domains in smectic due to chirality. (**c**) Focal conic domain viewed with bright-field microscopy and (**d**) the letter H focused by it, 43 μm above the FCD plane. (**e**–**g**) Circular cholesteric domain, (**e**), in bright-field microscopy with (**f**) the letter H focused by it, 50 μm above the lens plane, and (**g**) viewed between crossed polarizers. (**h**) Focal length vs. temperature. Points connected by lines represent experiments where the sample was cooled down from the cholesteric into the smectic phase. The transition temperature between cholesteric and smectic is indicated. Colors represent different concentration of chiral dopant.

Below 31 °C, 8CB is in the smectic phase, and round toric FCDs form reliably in the microchannels (Figure 2a). However, chiral dopant partly disrupts the FCDs (Figure 2b), as the smectic phase is incompatible with twist deformation, and this is especially evident in the smaller channels. We choose to focus on the 40 μm channels, which can accommodate well-formed, round FCD with diameter ranging from 10 to 30 μm in their center, which work as lenses (Figure 2c,d). FCDs are erased by heating above the smectic-nematic transition temperature. The focal length depends on the diameter of the FCD [27]. Uncertainty in focal length measurements is due to a combination of effects, as the images are sometimes blurry as a consequence of some irregularity in the FCD structures. In the presence of large arrays of FCDs of similar size, we can determine their average focal length from visual inspection. Uncertainty is also attributed to drift in the heating stage, which due to thermal expansion is up to 5 μm over 10 °C. Even with these caveats, it is possible to measure and compare the focal length of FCDs for mixtures of 8CB and chiral dopant at various concentrations and test their stability against temperature changes. Our results indicate that, within the explored range of chiral dopant concentration, the average size and the focal length of the FCDs do not change significantly, and that the focal length remains constant throughout a temperature range between 23 °C and the smectic-nematic transition.

In the cholesteric phase, under certain conditions, a variety of circular cholesteric domains appear (Figure 2e–g), which have lensing ability on par with FCDs (Figure 2f, Supplementary Video V1). These domains are stable with regards to small temperature fluctuations and are nearly perfectly circular. Importantly, they frequently occur at the same location as FCDs upon heating, with a central +1 topological defect pinned in the channel through the phase transition. These structures resemble cholesteric defects such as torons [3,4] or toron-like [5,6] structures, which can form in the presence of homeotropic anchoring. Measuring the focal length as a function of temperature, as the temperature is decreased from the cholesteric to the smectic phase, we notice that the lenses show a discontinuity between the two phases. Here, the domains become unstable right before the smectic transition, and the director field changes. The variation of focal length with temperature (Figure 2h) demonstrates that these lenses can function as microlenses with tunable focal length. While the focal length of the FCD microlenses remains constant in all the temperature range of the smectic phase, the focal length of the cholesteric domains changes with temperature. Preliminary measurements suggest that the relative change in focal length at the phase transition is higher than the relative change of cholesteric pitch at the phase transition, which is approximately 25%. However, it is clear that temperature affects the size of defects, and this in turn creates the variation in focal length. With this stratagem, we can create a system where both temperature-dependent and temperature-independent lenses exist. We test this behavior on mixtures with different concentrations of chiral dopant. Further investigation is needed to fully understand the variability of the lenses’ focal length in the cholesteric phase, but it is clear that chiral dopant affects the average size and the focal length of the cholesteric circular domains (unlike the FCDs), as shown in Figure 2h.

### 2.2. Defect Formation

**Figure 3:**
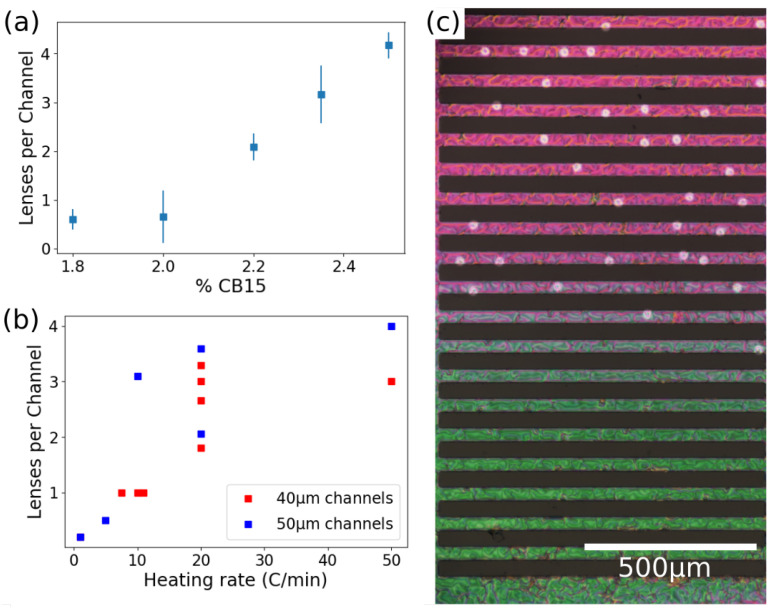
Formation of circular domains. (**a**) Number of circular domains for various concentrations of chiral dopant. (**b**) Number of circular defects formed as a function of heating rate in channels 40 and 50 μm wide. Every point corresponds to one experiment, in which the system was heated up to 36 ± 1 °C (far beyond the transition temperature) and in which the domains were counted. (**c**) Polarized microscopy image of the channels in the cholesteric phases. The circular domains can be clearly identified as brighter clear circles. It is possible to notice how the channels with the thinner layer (top) have more circular domains.

While cholesteric lenses demonstrate remarkable stability, their formation requires several conditions to form. The population of cholesteric lenses increases with the concentration of chiral dopant, as seen in Figure 3a. These defects occur at all concentrations tested but nearly double in population at higher concentration. However, this increase in population does not occur without any penalty. FCDs tend to be more distorted at higher chiral dopant concentrations (as seen in Figure 2b) and this behavior is especially prominent at 2.35% chiral dopant and above. This suggests that the optimal concentration of chiral dopant in order to maximize lens formation in both phases is between 2% and 2.2%.

Like chirality, heating rate also affects the chances of forming the chiral circular domains. We observed that cholesteric lens formation occurs only with large increase in heating rates when transitioning from the smectic to cholesteric phase (Figure 3b). Heating rates below 10 °C/min produce only sporadic cholesteric lenses at any CB15 concentration. This indicates that these structures are not the lowest energy state but are kinetically trapped when a fast temperature quench occurs.

Finally, the third crucial condition for stable cholesteric lens formation is the height of fluid in microchannels. We have observed consistently that in channels with a higher depth of fluid it is impossible to observe the cholesteric circular domains. This dependence on fluid height was first determined indirectly by the variation in color of transmitted light as seen in Figure 3c, which corresponds to a variation in film thickness according to the Michel–Levy color chart [28]. This variation in fluid height corresponds to a variation in microchannel height, which was measured by creating a poly-dimethyl-siloxane copy of the microchannels and inspecting the cross-section under an optical microscope. Circular cholesteric defects only form in channels with height ranging up to 13 μm and not in those taller than 14 μm. Thicker films also correspond to larger smectic FCDs, as the height and the diameter are related. Larger FCDs are more prone to being distorted by the chiral dopant, or to form irregular structures pinned at the edges of the channels, therefore the fluid has a maximum height which can accommodate the formation of both circular defects and FCDs.

### 2.3. Geometric Memory

**Figure 4:**
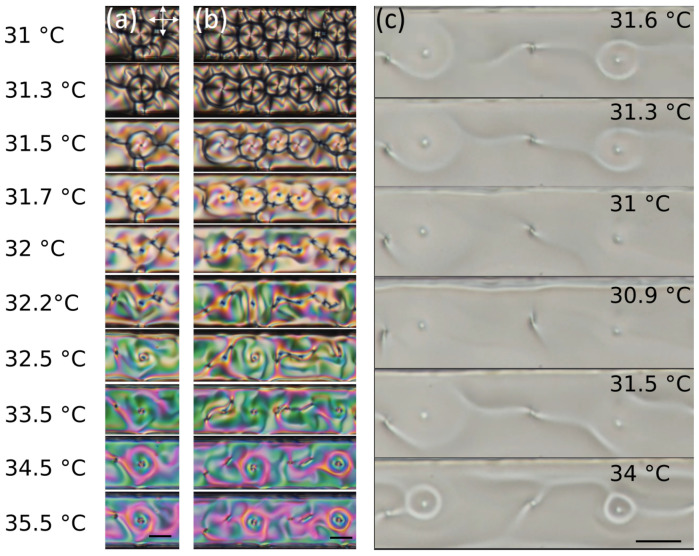
Memory at the smectic-cholesteric transition. (**a**,**b**) Two sequences of images from details of the supplementary video V2 showing defects heated up from smectic to cholesteric, with temperature ranging from 31 to 35.5 °C. It is possible to see how memory is retained at the phase transition. (**c**) Sequence of images from supplementary video V3 showing a circular cholesteric domain. The domain is cooled near the phase transition down to 30.9 °C (it is possible to see the “unwinding” of the cholesteric helix) and then heated up again to 34 °C before it turns into smectic, retaining its defect structure. All scale bars are 20 μm.

So far, we have focused on the optical properties of the lenses and the factors that allow for the formation of lenses in the first place. However, one key point is how to obtain lenses that remain in the same location and change characteristics at the smectic-nematic phase transition. Fortunately, the memory retained at the transition, which has been described between smectics and nematics in [25,26], also exists between smectics and weakly-chiral nematics. Cooling into the smectic phase, the circular cholesteric domains have a high probability of turning into FCDs, demonstrating the memory effect between smectic and nematic defects that has been seen in achiral liquid crystals (Figure 4a,b, Supplementary Video V2). The effect becomes prominent at higher chirality, above 2% chiral dopant. Despite the fact that the FCDs and the cholesteric defects have very different director configuration, the presence of the defect of topological charge +1 (radial alignment) in the cholesteric domain induces the formation of a FCD. Therefore, there is a high probability that cholesteric lenses turn into smectic lenses upon cooling. In this process, the structure of the defect core is likely to play an important role. There may be a variability in the core structure of circular cholesteric domains that is able to explain why only some of them turn into FCDs, as occurs at the nematic-smectic transition [25]. The reverse process, i.e., the transition from FCD to circular cholesteric domains upon heating, occurs less frequently. This is due in part to the difficulty in forming circular cholesteric domains, and in part to the strong rearrangement and deformations occurring at the phase transition from a more solid-like to a more liquid-like phase. Finally, also in this case the defect core structure of the FCD may be responsible: in fact, we have observed that FCD defects often tend to split into two defects with half-integer topological charge, which may prevent their ability to form circular cholesteric domains.

Our data show that a higher chiral dopant concentration increases not only the number of observed circular cholesteric domains, but also their stability. Right at the phase transition, the FCDs have the potential to form cholesteric lenses, but the majority of these defects are immediately annihilated with other defects or they move in the channel. An example of the improved stability of the cholesteric domains at concentrations higher than 2.35% is shown in Figure 4c and Supplementary Video V3. This shows cholesteric lenses that are cooled down and then re-heated. The size and focal length of the defects increase as the sample is cooled, but, when heated, these lenses return to nearly their original shape and do not disappear. This behavior is observed only at and above 2.35% chiral dopant. At or below 2.2%, cholesteric lenses cooled in this manner lose their structure and do not reform.

The kinetic pathway between FCDs and cholesteric circular domains still remains to be elucidated and will require further investigation, but our observations all suggest that kinetic trapping plays a major role in the formation of the circular domains. This is supported by the fact that, whenever the FCDs are able to relax to a more stable configuration, the lenses are not retained at the phase transition. We hypothesize that memory effect between smectic and cholesteric defects occurs because the defects are topologically protected. Furthermore, similarly to what is seen in [25], when the system is cooled the +1 defects in the cholesteric phase become the center of FCDs; when it is not cooled completely the director field can be recovered upon heating, as seen in Figure 4c. However, when cooled deeply into the smectic phase, the ability to recover the initial shape is lost, which also agrees with our experiments.

## 3. Discussion and Conclusions

We have shown how it is possible to utilize a liquid crystal with a small amount of chiral dopant to create tunable and reconfigurable micro-lenses using either smectic focal conic domains or circular cholesteric domains, and how it is possible to transform one type of defect into the other at the phase transition. The lenses are temperature-independent in the smectic phase, but they show a temperature dependence of the focal length in the cholesteric phase. The focal length is determined by the radius of the domain, which depends on the helical pitch and therefore changes with temperature and with the amount of chiral dopant. The formation of circular cholesteric domains is favored by adding more chiral dopant; however, an excess of it causes the disruption of smectic focal conic domains. Therefore, there is only a limited range of chirality where both types of defects can exist. Moreover, the circular cholesteric domains are only formed by fast heating from the smectic phase and are only formed in thin fluid layers. Finally, we have shown how one defect can turn into the other at the transition, and how a central topological defect can protect such transformation. This work sheds new light on the process of memory at the phase transition and offers an example of how such memory can lead to the preservation of defects and can be used to create new LC-based technology.

## 4. Materials and Methods

To prepare the material used for observations, the thermotropic liquid crystal 4-cyano-4’-octylbiphenyl (8CB, MilliporeSigma, St. Louis, MO, USA) is mixed with the chiral dopant 4-(2-methylbutyl)-4-cyanobiphenyl (CB15, Grand In Chem.). Several mixtures are prepared, ranging in concentration from 1.5% to 2.5% CB15 by mass. These mixtures have a smectic phase and a chiral nematic phase and the transition temperatures vary from 30.8 to 31.2 °C depending on the concentration of CB15. At these concentrations of chiral dopant, the cholesteric pitch ranges from 6.5 to 9.5 μm [29]. Mixtures are heated and vortexed before each use. In order to test the effect of temperature on the chiral pitch of the LC, we have measured uniform samples with homeotropic anchoring and observed the shift in the spacing of the fingerprint texture. We thus estimate the change in pitch to be Δp/p≈0.25.

**Figure 5:**
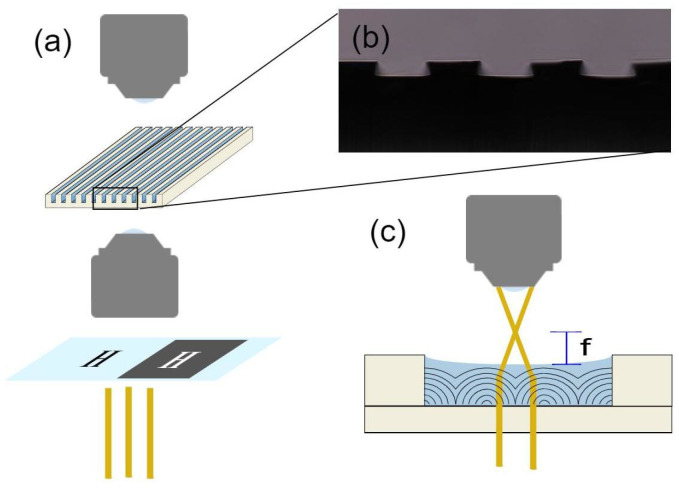
Experimental setup. (**a**) Schematic of the setup used to test lensing ability of LC defects in the microchannel array. A mask made out of marker (hand-drawn letter H) on a glass slide, or a paper cutout of the letter, is placed in front of the light source behind the condenser, then the sample and the formed image are viewed through an upright microscope. (**b**) Cross-section of a poly-di-methyl-siloxane (PDMS) mold showing the profile of the micro-fabricated channels, viewed under an optical microscope. (**c**) Schematic depicting image formation of the mask by a lens. The sample is adjusted vertically until the image is in focus at a distance *f* above the sample.

Samples consist of microfabricated SU-8 (MicroChem, Westborough, MA, USA) channels created using photolithography, then filled with LCs (Figure 5a,b). Channels are designed to be 15 μm tall (which still results in a small variation in height as shown in Figure 5b) with various widths between 20 and 50 μm. Degenerate planar anchoring is provided by the untreated SU-8, while the LC-air interface has homeotropic (perpendicular) molecular anchoring. To fill the channels, LC is heated to the isotropic phase using a hot plate and pipetted. We always allow samples to sit for up to one hour in the isotropic phase before cooling to allow LC to equilibrate.

The samples are observed using bright-field microscopy with a Nikon Eclipse LV110N Pol equipped with a DS-Ri2 (Nikon, Tokyo, Japan) camera. A sample is placed on a water-cooled heating stage Instec HC S302 MK 2000 (Instec Inc., Boulder, CO, USA) using various heating and cooling rates. In order to see the lensing effects created by structures in the sample, we use ‘masks’ consisting of black poster-board with shapes cut out or glass slides with corresponding shapes inscribed using a dark marker. The masks are placed between the microscope lamp and the condenser and used to project images onto the sample, as shown in Figure 5a, then the focal length is estimated as shown in Figure 5c.

Simulations of the cholesteric phase are done minimizing the Landau–de Gennes free energy with a finite difference scheme on a regular cubic mesh (details can be found in [24]).

## Supplementary Materials

The following are available online at https://www.mdpi.com/1996-1944/13/17/3761/s1. Video V1: lenses from a cholesteric domain; Video V2: Focal conic domains at the phase transition; Video V3: Cholesteric domains cooled and re-heated near the phase transition.

## References

[1] Kovshev E.I., Blinov L.M., Titov V.V. (1977). Thermotropic Liquid Crystals and Their Applications. Russ. Chem. Rev..

[2] Ye P., Gu C. (2010). Optics of Liquid Crystal Displays.

[3] Algorri J.F., Zografopoulos D.C., Urruchi V., Sanchez-Pena J.M. (2019). Recent advances in adaptive liquid crystal lenses. Crystals.

[4] Kim S.U., Na J.N., Kim C., Lee S.D. (2017). Design and fabrication of liquid crystal-based lenses. Liq. Cryst..

[5] Musevic I. (2012). Integrated and topological liquid crystal photonics. Liq. Cryst..

[6] de Gennes P., Prost J. (1993). The Physics of Liquid Crystals.

[7] Frank F.C. (1958). On the theory of liquid crystals. Discuss. Faraday Soc..

[8] Chandrasekhar S., Ranganath B.S. (1986). The structure and energetics of defects in liquid crystals. Adv. Phys..

[9] Nikkhou M., Skarabot M., Copar S., Ravnik M., Zumer S., Musevic I. (2015). Light-controlled topological charge in a nematic liquid crystal. Nat. Phys..

[10] Serra F. (2016). Curvature and defects in nematic liquid crystals. Liq. Cryst..

[11] Brasselet E. (2012). Tunable Optical Vortex Arrays from a Single Nematic Topological Defect. Phys. Rev. Lett..

[12] Kim M., Serra F. (2020). Tunable Dynamic Topological Defect Pattern Formation in Nematic Liquid Crystals. Adv. Opt. Mater..

[13] Serra F., Gharbi M.A., Luo Y., Liu I.B., Bade N.D., Kamien R.D., Stebe K.J. (2015). One-Step Assembly of Reconfigurable Smectic Liquid Crystal “Compound Eye” Lenses. Adv. Opt. Mater..

[14] Kim J.H., Kim Y.H., Jeong H.S., Srinivasarao M., Hudson S.D., Jung H.T. (2012). Thermally responsive microlens arrays fabricated with the use of defect arrays in a smectic liquid crystal. RSC Adv..

[15] Popov P., Honaker L.W., Mirheydari M., Mann E.K., Jakli A. (2017). Chiral nematic liquid crystal microlenses. Sci. Rep..

[16] Kitzerov H.S., Bahr C. (2001). Chirality in Liquid Crystals.

[17] Beller D., Machon T., Copar S., Sussman D.M., Alexander G.P., Kamien R.D., Mosna R. (2014). Geometry of the cholesteric phase. Phys. Rev. X.

[18] Kleman M., Lavrentovich O.D. (2009). Liquids with conics. Liq. Cryst..

[19] Choi M.C., Pfohl T., Wen Z., Li Y., Kim M.W., Israelachvili J.N., Safinya C.R. (2004). Ordered patterns of liquid crystal toroidal defects by microchannel confinement. Proc. Nat. Acad. Sci. USA.

[20] Ackerman P.J., Trivedi R.P., Senyuk B., van de Lagemaat J., Smalyukh I.I. (2014). Two-dimensional skyrmions and other solitonic structures in confinement-frustrated chiral nematics. Phys. Rev. E.

[21] Schlotthauer S., Skutnik R., Stieger T., Schoen M. (2015). Defect topologies in chiral liquid crystals confined to mesoscopic channels. J. Chem. Phys..

[22] Kim Y.H., Gim M.J., Jung H.T., Yoon D.K. (2015). Periodic arrays of liquid crystalline torons in microchannels. RSC Adv..

[23] Guo Y., Afghah S., Xiang J., Lavrentovich O.D., Selinger R.L.B., Wei Q.H. (2016). Cholesteric liquid crystals in rectangular microchannels: Skyrmions and stripes. Soft Matter.

[24] Sussman D.M., Beller D.A. (2019). Fast, Scalable, and Interactive Software for Landau-de Gennes Numerical Modeling of Nematic Topological Defects. Front. Phys..

[25] Suh A., Gim M., Beller D., Yoon D. (2019). Topological defects and geometric memory across the nematic-smectic A liquid crystal phase transition. Soft Matter.

[26] Gim M., Beller D., Yoon D. (2017). Morphogenesis of liquid crystal topological defects during the nematic-smectic A phase transition. Nat. Comm..

[27] Designolle V., Herminghaus S., Pfohl T., Bahr C. (2006). AFM Study of Defect-Induced Depressions of the Smectic-A/Air Interface. Langmuir.

[28] Sorensen B. (2013). A revised Michel-Levy interference colour chart based on first-principles calculations. J. Mineral..

[29] Ko S.W., Huang S.H., Fuh A.Y.-G., Lin T.H. (2009). Measurement of helical twisting power based on axially symmetrical photo-aligned dye-doped liquid crystal film. Opt. Express.

